# Evidence of Animal-Assisted Therapy in Neurological Diseases in Adults: A Systematic Review

**DOI:** 10.3390/ijerph182412882

**Published:** 2021-12-07

**Authors:** María del Carmen Rodríguez-Martínez, Alba De la Plana Maestre, Juan Antonio Armenta-Peinado, Miguel Ángel Barbancho, Natalia García-Casares

**Affiliations:** 1 Department of Physical Therapy, Faculty of Health Sciences, University of Malaga, C/Arquitecto Francisco Peñalosa 3, 29071 Málaga, Spain; marrodmar@uma.es; 2 ClinicaPodofisio, Rincón de la Victoria, 30204 Málaga, Spain; albadelaplana9@hotmail.com; 3 Pathological Anatomy and Physical-Sports, Education, Department of Human Physiology, Human Histology, University of Malaga, 29016 Málaga, Spain; 4 Centro de Investigaciones Médico Sanitarias (CIMES), University of Malaga, 29010 Málaga, Spain; nagcasares@uma.es; 5 Instituto de Investigación Biomédica de Málaga (IBIMA), 29010 Málaga, Spain; 6 Department of Medicine, Faculty of Medicine, University of Malaga, 29016 Málaga, Spain

**Keywords:** animal-assisted therapy, neurological disease, stroke, multiple sclerosis, dementia

## Abstract

Background: In recent years, the possibility of intervening humans with animal-assisted therapy (AAT) has been growing due to numerous physical, psychological, and social benefits provided to humanity, enabling them to maintain or improve their quality of life. There exist different animals through which this therapy can be performed. The purpose of this systematic review will focus on the effects of AAT in several neurological diseases. Methods: The search of clinical trials was carried out in the PubMed, Scielo, Embase and PEDro databases. The selection of articles was made according to the different inclusion and exclusion criteria, incorporating those that approached neurological diseases to be reviewed. Results: Twenty-five clinical trials were identified, seventeen of which were finally included in the review. The results indicate that animal-assisted therapy (AAT) in different neurological diseases has many benefits in several areas, for example, in motor and physical ability as well as in mental and behavioural health. Conclusions: This systematic review provides occupational therapy practitioners with evidence on the use of activity based on animal-assisted therapy as a novel field of intervention that can complement other therapies and obtain benefits in different populations.

## 1. Introduction

From the Prehistoric age, animals have lived with people as loyal partners while improving their quality of life. The relationship between humans and animals has been demonstrated throughout history, i.e., prehistoric cave paintings of humans, wolves with dogs as their offspring after being domesticated for between 10 and 20 thousand years ago [1]. Horses have played a major role in the evolution of human society since the Bronze and Iron Ages. They have been crucial to warfare before the development of firearms [2], although the origins date to 460 before Christ, when Hippocrates applied riding on horseback as therapy for fighting against insomnia and improving muscular tonicity [3].

Therefore, animals have sustained humans in several aspects of daily life, including as guardians of the youngest children, accompanying the hunters or leading the herd, or by attending to blind people or people with other disabilities [4]. According to the International Association of Human–Animal Interaction Organization (IAHAIO), the inclusion of animals within a therapeutic environment has existed since the end of the seventeenth century [5]. As documented by Filan et al., “animals make humans feel better and serve as aids to communication, reaching those who show little response to other stimuli” [6], the best known being in the areas of assistance dogs for people who are deaf or blind, but there is an emerging field of data on the use of assistance dogs for people with post-traumatic stress disorders and other areas of mental health and dogs that detect cancers and seizures [7]. As a result, due to the strong link between humans and animals, this therapy has emerged by incorporating animals into the rehabilitation process.

Animal-assisted interventions (AAIs) refer to the development of entertainment, recreation, and motivation to improve quality of life, while in AAT, there is a targeted intervention performed by a health professional with clear goals that is directed to develop and/or improve social, physical, emotional and cognitive aspects of the people involved [8,9]. Animal-assisted therapy (AAT) can be defined as those methods that include animals in the prevention and treatment of human mental and physical pathologies [10]. We can also define it as a goal-directed intervention in which an animal that meets specific criteria is an integral part of the treatment process (AWMA. Animal-assisted interventions: definitions).

The methods of action under which AAT works are different and include: multisensory stimulation, imitation, physical contact, play, generation of the feeling of affection, and reinforcement of desired and stress reduction [11,12]. The most suitable animals for AAT are fish, cats, dogs and horses [13], although birds, rabbits, dolphins, pigs, chicken or any other that is prepared regardless of their size can also help [14,15]; however, it depends on the individual receiving the therapy. In addition, this therapy has the main objective that can be developed in groups or individually in order to help people find total well-being [16]. 

In this systematic review, we will focus on dogs and horses as animals involved in therapy. Dogs are especially beneficial in the field of medicine and are more involved for this activity or for assisted therapy [14]. Furthermore, the horse also stands out as a co-therapist because we can perform therapy in three ways: passive hippotherapy, using the horse’s pace (the patient does not have an active role in the activity as the therapist leads the rehabilitation exercises); active hippotherapy (implementation of neuromuscular exercises that are aimed at promoting muscle tone, coordination, postural control and balance) [17]; therapeutic riding (equino therapy teaching as a sport, in which the patient rules the horse, engages with it and turns into an active rider; step, jog, and gallop are worked according to the skills developed by the patient) [18]. 

Some research has shown that AAT with dogs causes an automatic relaxation response, including: reducing feelings of anxiety and loneliness in mental health and a reduction of blood pressure that improves cardiovascular health, which therefore reduces the intake of medications in the area of physical health [19]. Other research has indicated that a pet has significant health effects such as lowered risk factors for cardiovascular disease, i.e., coronary heart disease [20,21], higher chance of surviving myocardial infarction [22,23], and less need of physician services during stressful life events [24].

Conversely, horses also have numerous therapeutic effects. The first is to improve postural control through the improvement of balance and straightening reactions [25], especially in people with impairment of muscle patterns or delayed action of antagonist muscles (in people with a lack of modulation of muscle tone such as cerebrovascular diseases or multiple sclerosis) [26]. The second is to increase human gait since to ride horses helps in the experience movements that are similar to humans when walking, which allows automation of gait until it becomes a functional pattern [27]. The third is to standardize muscle tone which hippotherapy provides through the stimulus of co-contraction between agonist and antagonist muscles, and which improves reciprocal innervation during the riding process [28]. Finally, hippotherapy involves a range of emotions, interactions, and cognitive processes in addition to providing benefits in the social aspect [29].

Furthermore, if we study the physiological effects of human–animal interactions, we can find effects in different indicators. For instance, being involved in a positive interaction with a dog was found to be relaxing enough that it lowered stress levels (reducing cortisol levels and causing a significantly higher increase of oxytocin, which plays an important role in pair bonding, social affiliation, and trust in many species) [30,31].

For all these reasons, the aim of this systematic review is to inquire the benefits that animal-assisted therapy can bring in the field of neurology (focusing on the following diseases: dementia, stroke, spinal cord injury, Parkinson, epilepsy, amyotrophic lateral sclerosis and multiple sclerosis), since there is wide evidence that these disorders represent one of the most serious threats for public health [32], and to inquire to the clinical manifestations that often include loss of muscle tone, selective motor control and balance [33], spasticity, and impaired strength [34], which can be improved with the proposed therapy.

## 2. Materials and Methods

### 2.1. Search Strategies

**Figure 1:**
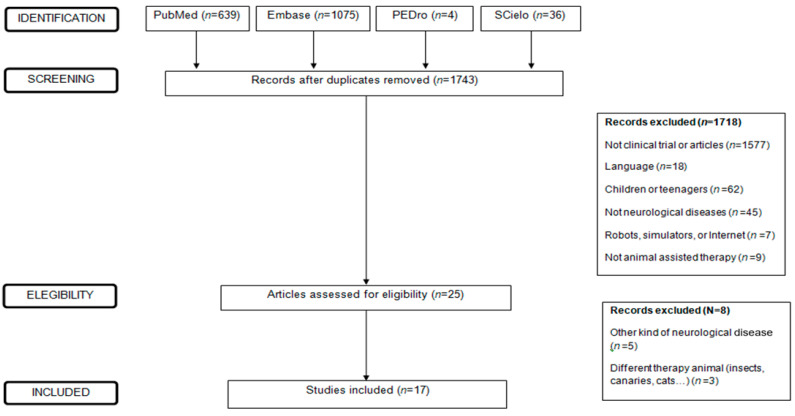
Flow of articles through the phases of the systematic review.

To carry out this systematic review, an exhaustive search was performed in the PubMed, Scielo, Embase and PEDro databases to identify clinical trials published until November 2020 without a chronological limitation in period of years. We also searched for systematic reviews and meta-analyses when this research started, and a search was previously carried out in PROSPERO in order to check if this topic had been developed, and no similar research had been found. On 23 November 2021, a new search was carried out, and there was no review that covered all the neurological diseases studied. Figure 1 was developed according to the PRISMA regulation, and the trial selection is attached (see Figure 1). The bibliographic search was performed until November 2020. The key search terms used were the same in all databases: (animal-assisted therapy) AND (neurological disease OR stroke OR multiple sclerosis OR spinal cord injury OR dementia OR Parkinson OR epilepsy OR ALSOR neuromuscular disease). Although the search was carried out in other neurological diseases such as amyotrophic lateral sclerosis (ALS), epilepsy, Parkinson’s, and neuromuscular diseases, no results were obtained for these pathologies.

### 2.2. Selection Criteria

Studies were included in this systematic review if they met the following criteria: study design was clinical trial, controlled clinical trial or randomized clinical trial; participants were adults, diagnosed with some neurological disease such as multiple sclerosis, stroke, dementia (Alzheimer’s), spinal cord injury, Parkinson, ALS, neuromuscular disease or epilepsy; each study had to detail a number of physical, mental or emotional points and then compare the results after the intervention with animal-assisted therapy (AAT); in the selected studies, it was necessary to specify the kind of test used to check both the changes produced after the intervention and the starting point of the patients that involved themselves.

Exclusion criteria were: letters to the editor, single case studies, communications to conferences, revision, or systematic review; not written in English or Spanish; performed on children or teenagers (in diseases such as cerebral palsy, autism…); no accurate diagnosis of neurological disease (for example, those aimed at the elderly, who did not have a diagnosis in dementia or those who were disorders of the psychiatric area, such as post-traumatic disorders); made in robots; other neurological diseases that were not addressed in the systematic review (acquired brain injury or minimally unconscious state); animal-assisted therapy not performed with horses or dogs solely (for example with canaries or insects).

The quality assessment of the selected studies was carried out by two independent researchers using PEDro Scale [35,36]. The scores of the studies are in Table 1. The mean score obtained according to the PEDro scale to assess the methodological quality of the clinical trials and pilot studies was 7/10.

## 3. Results

After the search, a total of 1743 results were obtained of which, after applying the inclusion and exclusion criteria, 17 clinical trials were selected (see Figure 1).

The results obtained are summarized in Table 1 (see Table 1). It includes: surname of the first author and year of publication of each study, neurological pathology that the clinical trial is about, type of study, number of patients, study design, characteristics of each intervention, measures (tests used in the study) and outcomes (results in summarized form of each clinical trial). Then, the articles were classified according to the neurological pathology that they address.

### 3.1. Results in Patients with Dementia

The patients submitted some type of dementia in seven studies [37,38,39,40,41,42,43]. Two of them were non-randomised. In particular, three articles of the seven mentioned [37,40,42] recruited patients with Alzheimer’s. Some diagnoses were based on the DSM-IV manual and the ICD-10. In all of them, the animals involved dogs in the therapy.

Different domains that may be affected in this neurological disease were measured with specific tests. Scales were used to assess depression symptoms in all studies except for one [39]. Except for one study [38], the rest used the MMSE scale to identify the cognitive state of the patients. In two of them [38,39], the quality of life in later phases of dementia was evaluated. Balance, emotional aspects and agitation were also evaluated [38,40,41,42,43]. 

Among the interventions made in these studies, we found: AAT adapted to reality orientation therapy, petting and greeting the dog, giving the dog treats and orders, brushing them, throwing a toy at them, brushing the dog’s teeth, dressing them, adjusting the collar, speaking with the dog, and on the dog to another person, taking a short walk. All of this was performed to stimulate sensory, social, cognitive, and motor functions.

Statistically significant results were found in all articles for the benefit of AAT, except for one [41], where in the intervention group, the results remained the same as at the beginning of the study. Among the most relevant results in patients with dementia where therapy was applied, the following stood out: decreases in stress levels via measured cortisol levels [37]; improvement in balance [38], improvement in symptoms of depression and quality of life [37]; significant results in physical, behavioural and emotional function [40]; increase of pleasure, motor activity and general alertness; reduced sadness [42]; and improvement of cognitive function [43].

### 3.2. Results in Patients with Multiple Sclerosis

Some studies were made in patients with multiple sclerosis and, in all, the animals that were involved for assisted therapy were horses [44,45,46]. One of them was a non-randomised comparative open study [44], another had previously published protocol [45], and the last was randomised [46]. 

The test used to evaluate the quality of life in all articles was MSQOL-54, which is specific to multiple sclerosis and to quality of life in patients with constipation; CVE-20 was specifically used. Fatigue was measured with FSS and FIS. The Asworth scale and the NRS were used to evaluate spasticity (the EDSS scale also evaluated it). Other aspects evaluated were general health perception (KHQ), depression (BDI), walking speed (T25-FW) and balance (BBS).

The interventions focused on different protocols. In study [44], the sessions were carried out by the equestrian team, which apart from including exercise on the horse’s back (sensory stimuli and different movements provided by the horse), carried out workshops of complementary activities such as cleaning the horse, field trips, attention to behaviour, and relationship with the environment (Terapias ecuestres) [47]. In study [45], patients received hippotherapy as defined by the German Board of Trustees for Therapeutic Riding (DKThR), where the horse is led by an assistant on a long rein and the physiotherapy treatment accomplishes the hippotherapist (Hippotherapie DKThR^®^) [48].

Statistically significant results were found in improving fatigue spasticity, balance, and pain perception [45]. There were also outstanding results in respect to the general perception of health and quality of life [44]. However, no significant results were obtained in depression, constipation, and gait.

### 3.3. Results in Patients with Stroke

Bunketorp-Käll et al. [49], Bunketorp-Käll et al. [50], Bunketorp-Käll et al. [51], and Beinotti et al. [52] all included stroke patients. Animal-assisted therapy (AAT) involved horses in all of them, and the studies were all randomised.

Bunketorp-Käll et al. studied the effects of AAT both on the aftermath of patients [47,48] and on the quality of life of their caregivers using the LISS questionnaire [51], the study protocol of which was previously published [53].

Among the study variables, balance (BBS, BDL-BS and BBL-BS scales), grip strength using the Grippit dynamometer, general cognitive level with the BNIS screen, and working memory with the LNS test were evaluated. To measure the impact of stroke, the SIS scale and quality of life scale were used with the SF-36 survey. In addition, upper limb function, stress, perception of physical functioning, perceived confidence, depression, self-efficacy, psychomotor speed, non-verbal learning, fatigue experienced, motor items, level of independence/dependence, and the quality and satisfaction of life were evaluated.

The interventions carried out were: preparation of the horse (grooming and equipping it), equestrian activity where the three-dimensional movements of the back of the horse caused sensorimotor experience similar to normal human gait, balance exercises (hands on head, making diagonals or circles), exercises of trunk rotation (touching different parts of the horse, using a stick), exercises to train affected parts (simulating riding a bicycle, lying with the arms around the horse, holding the reins...), cognitive exercises (giving instructions, participating in route planning), exercises to stimulate strength, relaxation exercises, body awareness, and deep, slow breathing.

Regarding the studies, significant results were obtained in the perception of recovery from stroke, remaining up to 6 months after the intervention [51], including increased SF-36 scores in the areas of functional capacity, physical aspects, and mental health [52]. In one of them, it was demonstrated that the quality of life was better in the caregivers of the patients when the horse-assisted therapy intervention was completed, including up to three months later (although it was not significant at 6 months) [50]. In the most recent study, the effectiveness of hippotherapy in improving gait and functional mobility was also observed, while in rhythm and music therapy, there were no such improvements evident [49].

### 3.4. Results in Patients with Spinal Cord Injury

Two studies of interest were found [54,55]. One of them was a quasi-experimental analysis and was performed in patients with spinal cord injury grouped into a single group [54]. The other was a crossover trial with three groups [55]. Assisted therapy was carried out with dogs in the first study and with horses in the second.

In the first study, the VAS and WUSPI scales were used. The first was to identify the level of effort perceived in the upper extremities, and the second was to identify shoulder pain in wheelchair users. In addition, the AIS scale was used to identify the sensory and motor severity of the patients. In the second, the VAS scale was also used. Other scales used were the Ashworth scale (for spasticity) and the Bf-S (to assess mental well-being).

In the quasi-experimental analysis, the intervention consisted of using a dog to climb a ramp. The dogs were previously trained for 5 months, and the users attended training classes. Significant results were obtained when climbing the ramp with the help of an assistance dog, with less effort made in the upper limbs (muscular use of the anterior deltoid, biceps and pectoral major was reduced) and greater speed [54].

In the crossover trial, twelve patients with spinal cord injury were divided into three intervention groups, rotating every four weeks with rest periods between the different treatments. One of the interventions was hippotherapy (HTK therapy, in which the subject was passively moved by the movement of the horse); in another, they performed therapy with the patients sitting astride a Bobath roll, and in the other, they performed with the users sitting on a stool with a rocking chair operated by an electric motor. In the short term, improvements in spasticity (clinically proven) were achieved in subjects who were treated with hippotherapy. In the self-reported spasticity by the VAS scale, there was no significant difference before and after each session in the intervention performed with the rocking chair. Instead, there were improvements in the other two therapies. Better mental well-being was also obtained after the horse therapy intervention, although in the long term, there were no improvements in any of the above aspects [55].

## 4. Discussion

This systematic review focuses on investigating the efficacy of animal therapy—in this case, dogs and horses—and in several neurological diseases (specifically spinal cord injury, stroke, dementia and multiple sclerosis).

In all the selected trials on dementia and spinal cord injury, animal therapy most commonly used dogs (except for one spinal cord injury article in which the horse was involved), whereas in those that recruited patients with multiple sclerosis and stroke, the most used animal therapy used horses. This suggests that working with one animal or another will enhance different effects in individuals; therefore, that it is necessary to choose which animal will be appropriate according to the disease or symptoms suffered by the user. It would be appropriate to highlight the different co-evolutions of the species involved, which implies different skills of the animals involved for therapeutic purposes. For this reason, attention should be given to interspecific relationships and the factors that influence them, as well as the interspecific relational skills of each individually chosen animal and its relationship with the owner, such as attachment styles and relational reciprocity between the animals, patients, and members of the working group [56].

The research revealed that dog therapy in people with dementia decreased stress [37], anxiety and sadness [42], and depressive symptoms [38,43], while increasing pleasure, general alertness [42] and improved behaviour [40]. On the basis of this information, it can be concluded that there is an improvement of symptoms at the psychological and cognitive level (in one of the studies, the score in the MMSE increased; thus, this therapy can be helpful in patients with great affectation of memory and reasoning, as well as in people with loss of social skills) [54]. Although, in one of the studies, the symptoms of depression, aggression and agitation remained constant in patients with severe and very severe dementias when combining animal-assisted therapy (AAT) with conventional treatment or therapy alone, and these symptoms increased over time [41]. This would suggest that animal therapy in such advanced dementias can maintain some of the symptoms, although not improve them; therefore, animals can help to stop their triggering, and the symptoms will not progress as quickly. This improves the quality of life in severe dementias, as evidenced by Olsen et al. in their studies [38,39]. Although the increase in quality of life is also closely related to improving balance, this animal therapy helps to improve the physical function and motor activity of the patients [40,42]. If we achieve an improvement in balance, there will be less risk of falls, and this will promote the well-being of our patients [38]. In respect to the improvement of agitation, there were no significant effects [38].

In users with multiple sclerosis, where the horse is also used as a co-therapist, an improvement in spasticity and general perception of health was observed, including a decrease in fatigue after 6 months of hippotherapy [44], although another study highlighted the same improvements in addition to an improvement in balance in a 12-week treatment, which was also combined with standard care [45]. This suggests that hippotherapy may be a great opportunity to improve the symptoms of patients with multiple sclerosis in terms of motor abilities, but it can be positive in relation to the bond with the horse due to the mixed emotions created by this therapy. In relation to patients with stroke, in the four studies carried out, the animal most involved in therapy was the horse. In two of them [49,51], whose protocol was previously published [53], apart from studying the efficacy of hippotherapy, the effects of music- and rhythm-based therapy were also investigated. In the first of them [51], it was shown that both therapies increased the perception of recovery in the late stages of stroke, and the results were lasted up to 6 months, while in hippotherapy, the score increased in the gait and balance tests, in rhythm and music therapy, and in grip strength and balance scores. This suggests that the combination of both could be effective. Therefore, it is important to consider the multimodal approach to adapt to each patient. The second study focused on the quality of life of caregivers of stroke patients [50]. It seems that it improves the quality of life of these informal caregivers, which is beneficial since, if we reduce their stress and anxiety about giving proper attention to their family or friends, better results will be achieved in these patients. In the most recent article, improvements were obtained in functional task performance after intervention with horses [49]. Conversely, Beinotti et al. [52] had already shown that hippotherapy-combined physiotherapy improved the quality of life of patients and produced a great improvement in physical aspects such as balance due to the properties of horse therapy. In addition, all these advantages were added to the challenge, in which users faced the emotions caused by this therapy, gained self-confidence, and felt more independent [52].

Finally, in patients with spinal cord injury, one of the articles mentioned dogs as assistants to the user in climbing a ramp. This helped to reduce mechanical load and muscle demand in the upper limbs [54]. This assistance can be beneficial such that shoulder pathologies and other pain do not occur in patients, but it can cause disadvantages in the dog’s health, since in the long term, it can cause problems such as hip dysplasia. For this reason, perhaps this therapy can be more harmful to the animal if it is not sufficiently trained and if it is subjected to too much working time. To an extent, we should not allow all the work to be performed by the animal unless the user is not suitable to perform a job or move around. Conversely, in another study with three different interventions (one of them was hippotherapy), improvements in spasticity and mental welfare were demonstrated with horse-assisted therapy compared to the other two therapies (based on stretching and passive rhythmic mobilizations) [55]. This shows the idea that the horse helps users with spinal injury in motor aspects such as spasticity due to movements that imitate human gait and helps users with the heat from the animal. Hippotherapy also promotes better mental health, and this may be because it is a novel therapy for many users and with it, they can discover various positive emotions. Although only short-term benefits are obtained, more evidence is needed to know how to work with these animals to favour long-term effects, since in this study, only 4 weeks of horse therapy was carried out due to the form of the trial. Thus, it would be important to conduct longer studies to check the effectiveness of long-term hippotherapy in patients with spinal cord injury.

In order for all these effects to be produced, it is important for the love and affection that an animal gives to be mutual. As Iannuzzi and Rowan point out: “Reconciling animal risks with their rehabilitative value is not simple or easy, unless we follow the principle that animals should not be used at all as a means to an end” [57]. Although a study showed (using serum and salivary cortisol levels, rectal temperature, blood pressure, systolic pressure, and resting heart and respiratory rates before, after, and 24 h after therapy) that the therapy did not seem to cause significant stress for dogs [58], animals need their rest periods such that their health is not adversely affected [59]. It is therefore necessary to consider the ethical aspects of an AAT program and to establish a basis of non-exploitation in which human beings are the ones who choose the type of relationship [59]. Among these principles are:

- Animals must be protected from abuse, discomfort, and stress, from a physical and mental point of view;
- Animals must be provided with adequate health care;
- Animals must have access to a quiet place where they can spend time away from their activities;
- Interactions with users should be structured such that the animal’s ability to serve as a therapeutic agent is maintained;
- A situation of abuse or stress will be never allowed (in this case, the session would be suspended immediately).

Therefore, it is essential that guidelines be established in which the highest priority is respect for the animals, because it is impossible to establish a quality relationship that helps the patient if it causes stress to the animal, wasting the great help they can give through the application of therapy. As a consequence, there would be no positive effects to the patient.

In 2015, the Italian Ministry of Health published a document on this topic, explaining the need for safeguards and monitoring the well-being during treatment sessions and periods of inactivity through clinical and behavioural visits. Any physical, physiological and/or behavioural changes must be registered. The veterinarian or the coadjutor of the animal in need must order the interruption of the intervention. Animals which, for any reason related to age or their health condition, are no longer involved in AAT must be ensured an adequate living condition [60].

Regarding zoo sanitation and sanitary controls that must be carried out to avoid the risk of zoonosis during IAA, studies have been increasing in this last period. Murthy et al. [61] provided some recommendations for the safe oversight and management of animals in healthcare. It should comply with legal requirements and minimize the risk of transmission of pathogens from animals to humans when animals are permitted in the healthcare setting [61]. In this way, it is important to collaborate between veterinarians, physicians, public health operators, and epidemiologists in order to prevent the transmission of these bacteria and achieve optimal health for humans, animals and the environment [62].

### Limitations

There are heterogeneous studies in methodology (in terms of study duration, sessions, and type of animal). The samples are small and there are few studies; thus, longitudinal studies are needed. Mental health aspects have not been studied in depth nor has psychiatric disease been included, as this may be the subject of another study.

## 5. Conclusions

In conclusion, it would be appropriate to mention that, apart from the multiple effects in the field of adult neurology, assisted therapy with dogs and horses is also present as a treatment option in many other pathologies and fields. For instance, in respect to studies with dogs, a decrease in stress levels and improvement in social communication were observed in adults with autism-spectrum disorders [63]; in patients and staff of a burn centre, the implantation of a therapy program with dogs caused a better mood after seeing them and both staff and patients were satisfied with this method [64]; in hospitalized patients awaiting a heart transplant, therapy was recognized as a general positive complement to usual medical care and a contributor to the increase in the perceived quality of hospitalization [65]; there was a development of the human–animal bond in a psychiatric prison that supported inmates’ correctional plans through greater recognition of their feelings and emotions [66]. Conversely, we found that there are also great beneficial effects in horse therapy. Among them, we find that hippotherapy can be a potential treatment to help improve gross motor function in children with Down syndrome [67], to improve attachment in adolescents with Internet gaming disorders [68], to decrease pain and to improve range of movement and quality of life for adults and older adults with arthritis [69], to change from moderate to vigorous physical activity and have greater self-efficacy for physical activity, and to be a pleasant treatment in childhood obesity [70].

All these results, as well as in neurological diseases as mentioned in the previous paragraph and in other areas (such as prisons), suggest that animal-assisted therapy (AAT) may be beneficial in the cognitive, social and physical areas. The beneficial effects deriving from AAT should also consider the psychological component linked to the co-evolution of dogs with humans [71,72], but further research with more samples and duration is necessary to consolidate and establish more evidence in this regard, such that there are not many contradictions in addition to carrying out studies in other types of neurological diseases in which therapy has not been tested and could have beneficial effects on such as Parkinson’s or ALS, although this may greatly depend on the type of person to whom the therapy is directed (there are people who are afraid of animals and can be counterproductive), the animals involved and the characteristics they have (therefore it is necessary to be properly trained), and the person leading the therapy. Another important point is to achieve greater dissemination such that there are more health professionals trained in this therapy and to offer it as an optimal treatment alternative. It is important to consider that pets are a consistent source of attachment security; future research with attachment measures may be useful for understanding how the relationship with a pet affects other aspects of the owner’s life, perhaps by buffering the experience of negative human social interactions [73]. The design of integrated studies is necessary to ensure that added value can be achieved in terms of synergistic benefits between the person and the pet during animal-assisted interventions. More specific guidelines for human–animal interaction should be developed based on such evidence-based knowledge [74].

In addition, there are studies that report decreased stress in dogs when working in the presence of their human referent [75].

## Figures and Tables

**Table 1:** The outcomes reported in research papers selected for systematic review.

Study Design								
First Author and Year	Neurological Pathology	Type of Study	Participants (*N*)	Study Group	Characteristics of the Intervention (Duration and Type of Animal)	Measures	Outcomes	PEDro Score
Bunketorp-Käll et al. (2019) Part of clinical trial of Bunketorp-Kall et al. (2012)	Stroke	Randomised Longitudinal	*N* = 123	Patients with stroke in late stage subjected to therapy based on rhythm and music (*N* = 41) vs. stroke patients subjected to hippotherapy (*N* = 41) vs. control group (*N* = 41)	12 weeks (twice per week) Horses	10 mWT; 6 MWT; M-MAS UAS	The group receiving hippotherapy completed the 10 mWT test faster compared to the control and had improvements in functional task performance. The effectiveness of music and rhythm therapy is less in the areas where it was improved with hippotherapy	8/10
Menna et al. (2019)	Dementia (Alzheimer’s disease)	Randomised Longitudinal	*N* = 22	Alzheimer’s patients subjected to AAT (*N* = 11) vs. control group (*N* = 11)	3 months (12 meetings, once per week) Dogs	MMSE; GDS	Decrease in cortisol level in the group treated with TAA	8/10
Muñoz-Lasa et al. (2019)	Multiple sclerosis	Non-randomised comparative open Longitudinal	*N* = 10	Patients with multiple sclerosis subjected to hippotherapy (*N* = 6) vs. control group (*N* = 4)	6 months Horses	T25-FW; Modified Ashworth Scale; BDI; MSQOL-54; FIS; KHQ; CVE20	Statistically significant results in terms of spasticity, fatigue, general perception of health and quality of life. No significant results in: T25-FW, depression, constipation.	6/10 8/10
Bunketorp-Käll et al. (2018) Use of Bunketorp-Käll et al.study (2017)	Stroke	Randomised Longitudinal	*N* = 106	Patients with stroke in late stage subjected to therapy based on rhythm and music (*N* = 37) vs. stroke patients subjected to hippotherapy (*N* = 37) vs. control group (*N* = 32)	12 weeks (twice per week) Horses	LISS	The LISS score changed when animal intervention was completed, and three months later, it was significantly higher in the caregivers in the intervention group than in the control group (especially in the ‘concerns’ section). It was not significant at six months.	
Vermöhlen et al. (2018) Continuation of Wollenweber et al. study (2016)	Multiple sclerosis	Randomised Longitudinal	*N* = 70	Multiple sclerosis patients who received hippotherapy plus standard treatment (*N* = 32) vs. control group (*N* = 38)	12 weeks (once per week) Horses	BBS; FSS; NRS; VAS; MSQOL-54; EDSS	Balance improved in both groups (most notably in the intervention group). Fatigue and spasticity improved in the intervention group and did not change in the control group. Pain perception improved in both groups.	9/10
Bunketorp-Käll et al. (2017) Continuation of Bunketorp et al. study (2012)	Stroke	Randomised Longitudinal	*N* = 123	Patients with stroke in late stage subjected to therapy based on rhythm and music (*N* = 41) vs. stroke patients subjected to hippotherapy (*N* = 41) vs. control group (*N* = 41)	12 weeks (twice per week) Horses	SIS; TUG; BBS; BDL-BS; Grippit (dynamometer); BNIS; LNS	Better results in the perception of recovery from stroke with rhythm and music therapy and horse-riding therapy than in the control group (they were maintained 6 months later).	8/10
Wollenweber et al. (2016)	Multiple sclerosis	Randomised Longitudinal	*N* = 70	Patients with multiple sclerosis subjected to hippotherapy as a complement to physiotherapy and pharmacotherapy (*N* = 35) vs. control group (*N* = 35)	12 weeks (once per week) Horses	BBS; FSS; MSQoL-54; VAS; NRS	Study protocol on the effectiveness of hippotherapy in the symptoms of multiple sclerosis	5/10
Olsen et al. (2016)	Dementia	Randomised Longitudinal	*N* = 80	Patients with dementia subjected to animal-assisted activity (*N* = 42) vs. control group (*N* = 38)	12 weeks (twice per week) Dogs	MMSE; BBS; QUALID	Balance improved in patients in the intervention group but was not statistically significant three months later. No effects on the quality of life of these patients were found.	7/10
Martín-Lemoyne et al. (2016)	Spinal cord injury	Quasi-experimental analysis Cross-sectional study	*N* = 10	Upper limb effort is quantified in patients with spinal cord injury when climbing a ramp with a wheelchair with and without the help of a mobility assistance dog.	Dogs	VAS; WUSPI; AIS	The involvement of an assistance dog allowed the speed when climbing the ramp to be significantly higher and allowed less effort from the upper limbs. Relative muscle utilization (anterior deltoids, biceps, and pectoralis major) and perceived effort in the upper limbs were significantly reduced.	5/10
Friedmann et al. (2015)	Dementia (Alzheimer’s disease)	Randomised Longitudinal	*N* = 40	Alzheimer’s patients subjected to assisted dog intervention (*N* = 22) vs. comparison group (*N* = 18)	12 weeks (twice per week) Dogs	MMSE; AES; CSDD; CMAI; Barthel Index	Pet therapy had statistically significant results on physical, behavioural, and emotional function in patients. There were no significant differences in terms of medication use.	7/10
Majic et al. (2013)	Dementia	Randomised Longitudinal	*N* = 65	Patients with dementia subjected to animal-assisted activities (*N* = 30) vs. control group (*N* = 35)	10 weeks (once per week) Dogs	MMSE; DSM-IV; CMAI; DMAS	Symptoms of depression and agitation were exacerbated in the control group. In the intervention group, they remained stable.	7/10
Beinotti et al. (2013)	Stroke	Randomised Longitudinal	*N* = 24	Patients with stroke subjected to horse-riding therapy (*N* = 12) vs. control group (*N* = 12).	16 weeks (three times per week) Horses	SF-36; DSM-IV	The SF-36 score increased in the intervention group (in the areas of functional capacity, physical aspects and mental health, and no significant differences were shown in the subdomains of general health, vitality, and emotional aspects). The SF-36 score decreased in the control group.	8/10
Bunketorp-Käll et al. (2012)	Stroke	Randomised Longitudinal	*N* = 120	Patients with stroke in late stage subjected to therapy based on rhythm and music vs. stroke patients subjected to hippotherapyvs. control group	12 weeks (twice per week) Horses	ICF; SIS; ADL; ARAT; BBS; BBL-BS; BBT; BNIS; EQ-5D; FIS; FES; GSES; LISS; Lisat-9; MADRS-S; M-MAS-UAS; NVLT; Ruff 2 and 7 SAT; SIS; SOC; TAP; TUG; VAS; WAIS; 6MWT; Grippit; Abilhand	Study protocol to assess whether rhythm and music therapy and TAA are effective in patients in the late stroke phase.	5/10
Mosello et al. (2011)	Dementia (Alzheimer’s disease)	Non-randomised Longitudinal	*N* = 10	1. Habitual day-care activity 2. Control activity with stuffed animals 3. Assisted animal activity	2 weeks before the intervention, 3 weeks of control activity with stuffed dogs, and 3 weeks of assisted activity with animals. Dogs	MMSE; SIB; ADL; CSDD; CMAI; NPI; OERS; ABMI; MoBOF	Significantly increased pleasure, motor activity and general alertness; and sadness decreased (three hours later) compared to the control group. The CMAI and CSDD scores were not significant.	5/10
Moretti et al. (2011)	Dementia	Non-randomised Longitudinal	*N* = 21	Patients with dementia subjected to animal therapy (*N* = 10) vs. control group (*N* = 11)	6 weeks (once per week) Dogs	MMSE; GDS; ICD-10	Decrease in GDS score in both groups. In the MMSE, they were not significant. Improvement in cognitive function in people who had animal therapy.	7/10
Lechner et al. (2007)	Spinal cord injury	Randomised Crossover trial	*N* = 12	The patients were divided into three groups: 1. Intervention H: hippotherapy 2. Intervention R: sitting astride a Bobath roll 3. Intervention S: sitting on a stool with a rocking chair. Every 4 weeks the intervention was changed	4 weeks (twice per week) each intervention with rest period between each Horses	Ashworth Scale; VAS; Bf-S	Hippotherapy reduced short-term spasticity and temporarily improved mental well-being. Sitting on a Bobath roll astride (stretching) or sitting on a rocking seat (passive rhythmic movements) did not have the same effects.	7/10

Abbreviations: MMSE: Mini-Mental State Examination; GDS: Geriatric Depression Scale; T25-FW: Timed 25-foot walk; BDI: Beck Depression Inventory; MSQOL-54: Multiple Sclerosis Quality of Life-54; FIS: Fatigue Impact Scale; KHQ: King’s Health Questionnaire; CV20: Quality of life in constipation; LSS: Life Situation among Spouses; EDSS: Expanded Disability Status Scale; FSS: Fatigue Severity Scale; VAS: Visual Analogue Scales; NRS: Numeric Rating Scale; SIS: Stroke Impact Scale; BDL-BS: Bäckstrand, Dahlberg y Liljenäs Balance Scale; QUALID: Quality of Life in Late-stage Dementia; BARS: Brief Agitation Rating Scale; AES: Apathy Evaluation Scale; CSDD: Cornell Scale for Depression in Dementia ; CMAI: Cohen-Mansfield Agitation Inventory ; DSM-IV: Diagnostic and Statistical Manual of Mental Disorders, Fourth Edition; CMAI: Cohen-Mansfield Agitation Inventory; SF-36: Medical Outcomes Study 36; ICF: International classification of functioning, disability, and health; ADL: Activities of Daily Living; ARAT: Action Reach Arm Test; BBS: Bergs Balance Test; BBL BS: Bäckstrand, Dahlberg, and Liljenäs Balance Scale; BBT: Box and Blocks Test; BNIS: Barrow Neurological Institute Screen for Higher Cerebral Functions; EQ-5D: EuroQol; FES: Falls-Efficacy Scale; GSES: General Self-Efficacy Scale; MADRS-S: Montgomery-Åsberg Depression Rating Scale–Self rate; M-MAS UAS: Modified Motor Assessment Scale according to the Uppsala University hospital; NVLT: Non-Verbal Learning Test ; Ruff 2 and 7 SAT: Ruff 2 and 7 Selective Attention Test; SOC: Sense of Coherence; TAP: Test for Attentional Performance; TUG: Timed Up and Go; WAIS: Wechsler Adult Intelligence Scale; 6MWT: 6-Minute Walk Test; 10mWT: 10 m Walk Test; CMAI: Cohen-Mansfield Agitation Inventory; NPI: Neuropsychiatric Inventory; OERS: Observed Emotion Rating Scale; ABMI: Agitated Behaviour Mapping Instrument; ICD-10: International Classification of Diseases, tenth Revision; WUSPI = Wheelchair User’s Shoulder Pain Index; CDR: Clinical Dementia Rating Scale; DMAS: Dementia Mood Assessment Scale; MoBOF: Motor Behaviour Observation Form; SIB: Severe Impairment Batter; AIS: American Spinal Cord Injury Association Impairment Scale; TUG: Timed Up and Go; LNS: Letter–number sequencing test; Bf-S: Befindlichkeits-Skala (well-being scale).
